# Genetic evidence links alcohol, coffee, cheese, and anxiety to GERD risk: A Mendelian randomization study

**DOI:** 10.1097/MD.0000000000047701

**Published:** 2026-02-20

**Authors:** Qingqing Zhang, Di Wu, Fengyun Guo, Shengnan Yang, Lijing Bao, Ruiying Zhang, Ping Wang

**Affiliations:** a Institute of Digestive Diseases, Xiyuan Hospital of China Academy of Chinese Medical Sciences, Beijing, China; b Graduate School of China Academy of Chinese Medical Sciences, Beijing, China; c First Affiliated Hospital of Heilongjiang University of Traditional Chinese Medicine, Harbin, China; d Graduate School, Beijing University of Chinese Medicine, Beijing, China.

**Keywords:** alcohol drinking, anxiety, cheese, coffee, gastroesophageal reflux disease, Mendelian randomization

## Abstract

In previous observational studies, an association has been found between the frequency of alcohol consumption, coffee intake, cheese consumption, and anxiety and the risk of gastroesophageal reflux disease (GERD). However, conflicting conclusions exist among these studies, and the causal relationship between these exposure factors and GERD remains unclear. Independent genetic variants associated with alcohol consumption frequency, coffee intake, cheese consumption, and anxiety at the genome-wide significance level were selected as instrumental variables. Summary-level data for GERD were derived from a genome-wide association meta-analysis that included 78,707 cases and 288,734 European-ancestry controls. The primary analysis methods were inverse-variance weighted (IVW), weighted median, MR-Egger, simple mode, and weighted mode methods serving as complementary approaches to IVW. Sensitivity analyses were conducted using Cochran *Q* test, MR-Egger intercept test, and leave-one-out analysis to assess the stability of the results. The IVW results demonstrated a strong positive causal relationship between the frequency of alcohol intake (odds ratio [OR] = 1.52, 95% confidence interval [CI] = 1.25–1.84, *P* < .001) and anxiety (defined as having consulted a general practitioner for nerves, anxiety, tension, or depression) (OR = 22.60, 95% CI = 12.12–42.15, *P *< .001) with GERD. A negative causal relationship was observed between genetically predicted cheese consumption and GERD (OR = 0.36, 95% CI = 0.26–0.50, *P *< .001). However, the association between coffee consumption and GERD was not significant in the IVW analysis (OR = 1.21, 95% CI, 0.98–1.60, *P* > .05). This study unveils causal connections between the frequency of alcohol consumption, cheese intake, and anxiety concerning GERD. Furthermore, our analysis found no strong genetic evidence to support a causal link between coffee consumption and GERD.

## 1. Introduction

Gastroesophageal reflux disease (GERD), characterized by the reflux of gastric contents into the esophagus, is a highly prevalent disorder affecting up to 20% of Western populations with a rising global prevalence.^[1,2]^ Clinically categorized into reflux esophagitis and endoscopy-negative reflux disease,^[3]^ GERD imposes substantial socioeconomic burden through frequent healthcare utilization while increasing the risk of complications, including Barrett esophagus and erosive tooth wear.^[4–6]^ The pathophysiology involves multifactorial mechanisms, such as lower esophageal sphincter dysfunction and delayed acid clearance, with emerging evidence implicating dietary and psychological factors in disease progression.^[7–9]^

Current epidemiological evidence regarding the risk factors of GERD remains controversial. Although frequent alcohol consumption,^[10]^ coffee intake,^[11]^ cheese consumption,^[12]^ and anxiety^[13]^ have been proposed to increase GERD risk through sphincter relaxation, conflicting studies have reported null or even protective associations.^[14]^ These discrepancies may stem from residual confounding and reverse causality inherent in observational designs. Notably, these exposures represent prevalent lifestyle factors, with anxiety affecting 4.3% of an individual’s lifetime,^[13]^ coffee/alcohol being widely consumed,^[15,16]^ and cheese constituting 45% of dairy intake in Western diets,^[17]^ underscoring the need for robust causal evidence.

Mendelian randomization (MR) analysis,^[18]^ which leverages genetic variants as instrumental variables, overcomes the key limitations of observational studies by minimizing confounding and precluding reverse causation.^[19,20]^ We implemented a 2-sample MR framework to systematically evaluate the causal relationships between modifiable exposures and GERD risk. Our findings provide high-quality evidence to inform the clinical guidelines and public health strategies for this pervasive disorder.

## 2. Materials and methods

### 2.1. Study design

**Figure 1.**
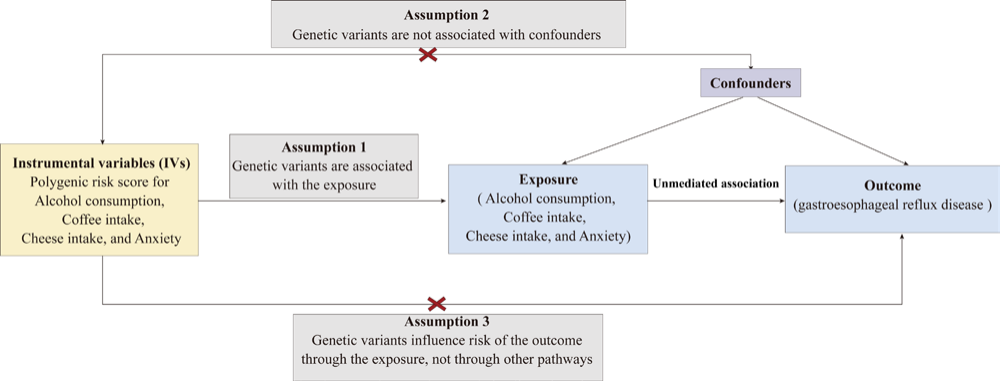
Construction of genetic instruments, data sources, and analysis plan for the relationship between alcohol intake frequency, coffee intake, cheese intake, and nerves, anxiety, tension, or depression with gastroesophageal reflux disease. SNP = single nucleotide polymorphism.

This MR study utilized rigorously selected single nucleotide polymorphisms (SNPs) derived from high-quality genome-wide association studies (GWAS) to examine the causal relationships between 4 modifiable exposures (alcohol consumption frequency, coffee intake, cheese consumption, and anxiety) and GERD risk. The validity of our MR analysis rests on 3 fundamental assumptions illustrated in Figure 1: 1st, the genetic instruments exhibit strong, well-documented associations with each target exposure (*P* < .001, *F*-statistic > 10); 2nd, that these SNPs influence GERD exclusively through their effects on the specified exposures without alternative pathways; and 3rd, that the variants remain independent of potential confounding factors affecting either exposures or outcomes. To ensure robust causal inference, we implemented comprehensive sensitivity analyses, including MR-Egger regression and weighted median approaches, which account for potential pleiotropy and strengthen confidence in our findings. This approach leverages the random allocation of genetic variants at conception to emulate a natural randomized controlled trial, thereby providing reliable evidence unconfounded by environmental or lifestyle factors that typically limit observational studies.

### 2.2. Genetic instrument selection and data sources

We derived genetic instruments for alcohol consumption frequency, coffee intake, cheese intake, and anxiety from a high-powered GWAS conducted in the UK Biobank cohort, with sample sizes ranging from 428,860 to 462,346 participants of European ancestry. For anxiety, the genetic instruments were sourced from UK Biobank field 2090, which records participants who have “seen a doctor (general practitioner) for nerves, anxiety, tension, or depression.” We employed this composite definition to capture a phenotype of significant clinical severity (that is, psychological distress substantial enough to prompt help-seeking behavior in a primary care setting). This definition is inherently broad, encompassing a spectrum of commonly co-occurring conditions in clinical practice, including generalized anxiety, depressive symptoms, and related somatic manifestations (e.g., “nerves,” “tension”). Instrument selection followed a stringent multistep protocol to ensure that the MR assumptions were met. First, we identified SNPs associated with each exposure at genome-wide significance (*P *< .001) with a minor allele frequency > 0.01. Using European reference panels, we performed LD clumping (*r*^2^ < 0.01 within 10,000 kb windows) to guarantee the independence of genetic variants.

**Figure 2.**
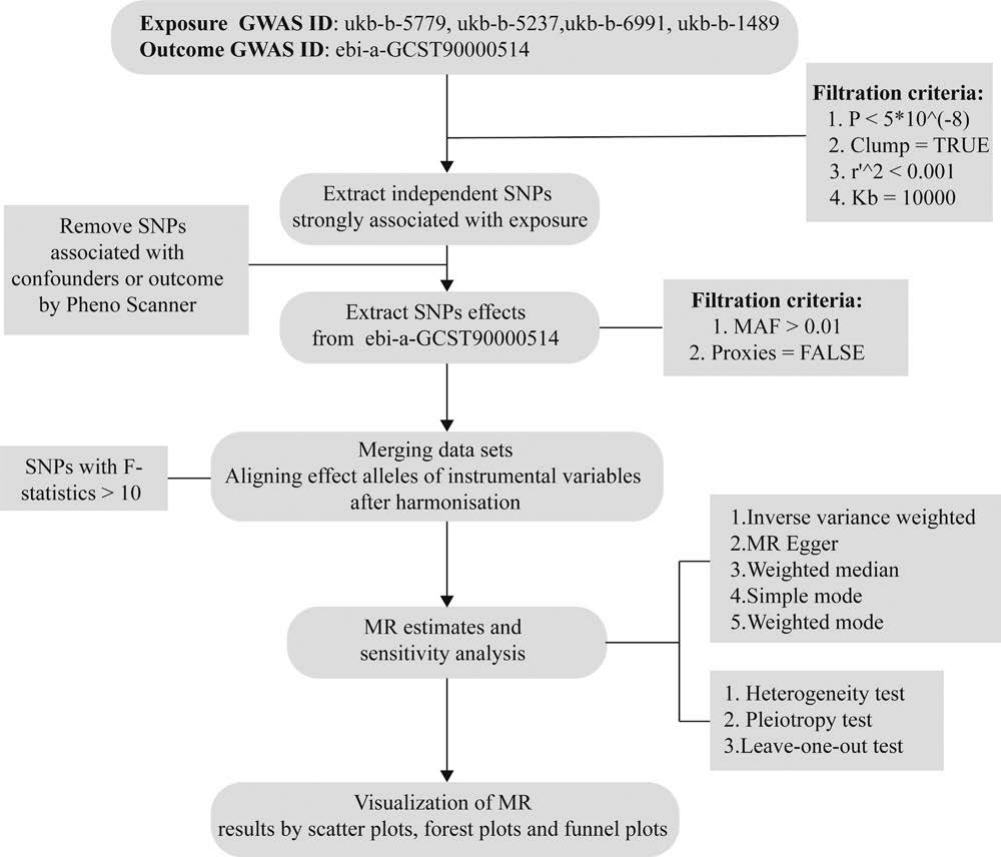
Study flowchart: GWAS = genome-wide association studies, MAF = minor allele frequency, MR = Mendelian randomization.

To mitigate weak instrument bias, we calculated *F*-statistics for all candidate SNPs, retaining only those with *F* > 10, indicating strong instruments (*F* = [(*N* - *k* - *1*)/*k*] × [*R*^2^/(1 - *R*^2^)], where *R*^2^ represents the proportion of explained exposure variance). We systematically evaluated potential violations of MR assumptions using the LDlink suite (https://ldlink.nih.gov/), screening, and removing SNPs associated with known confounders (Independence Assumption). The exclusivity assumption was verified through a comprehensive pleiotropy assessment using sensitivity analyses. The complete analytical workflow is illustrated in Figure 2.

### 2.3. GERD data sources

We utilized summary-level GWAS data from a large-scale meta-analysis combining 78,707 GERD cases and 288,734 controls of European ancestry derived from the UK Biobank and QSkin studies.^[21]^ The QSkin study,^[22]^ a longitudinal population-based cohort of Queensland residents, was originally established to investigate the etiology of skin cancer while collecting comprehensive lifestyle exposure data. The UK Biobank,^[23]^ comprising approximately 500,000 participants, provides extensive phenotypic and genotypic data with rigorous quality control measures.

This combined dataset offers several methodological advantages: enhanced statistical power through a large sample size; improved external validity via population-based sampling; and reduced population stratification bias through European ancestry restrictions. The GERD case definition incorporated a validated tripartite algorithm^[21]^ encompassing: ICD-10 code K21 diagnoses; proton pump inhibitor/H2 antagonist prescriptions; or self-reported physician-diagnosed GERD with regular acid-suppressing medication use.

### 2.4. MR analysis

We employed a 2-sample MR framework to investigate the causal relationships between 4 modifiable exposures (alcohol consumption frequency, coffee intake, cheese intake, and anxiety) and GERD risk. Our primary analysis utilized inverse-variance weighted (IVW) meta-analysis with random effects,^[24]^ which aggregates effect estimates across multiple genetic variants, while accounting for heterogeneity. Although IVW provides an efficient estimation when all instrumental variables are valid,^[25]^ its sensitivity to directional pleiotropy prompted us to implement complementary robust methods. MR-Egger regression^[26]^ addresses potential pleiotropic effects through its intercept term, relaxing the strict instrumental variable assumptions of IVW at the cost of reduced statistical power. For additional robustness, we apply the weighted median approach,^[25]^ which yields consistent estimates when at least 50% of the weight comes from valid instruments. Further refinement came from mode-based methods: the weighted mode estimator^[27]^ clusters variants by effect direction while weighting by precision and the simple mode groups SNPs by genetic loci to provide pleiotropy-resistant estimates. This multi-method analytical strategy balanced detection power with robustness against various forms of bias, ensuring a comprehensive evaluation of causal effects while characterizing potential violations of MR assumptions.

### 2.5. Sensitivity analysis

To ensure the robustness of our MR findings, we implemented a comprehensive sensitivity analysis framework. We 1st assessed instrumental variable heterogeneity using Cochran *Q* test,^[28]^ with *P* < .05, indicating significant heterogeneity and warranting random effects IVW estimation.^[29]^ Potential horizontal pleiotropy was evaluated using MR-Egger regression intercept analysis, where non-zero intercepts (*P* < .05) suggested directional pleiotropy.^[30]^ We systematically identified influential outliers via leave-one-out analysis, iteratively excluding individual SNPs and recalculating the IVW estimates to detect variants disproportionately affecting the results.^[31]^

Visualization played a key role in result interpretation: forest plots displayed effect size consistency across variants, scatter plots verified linear exposure-outcome relationships, and funnel plots evaluated for potential bias. These complementary graphical approaches provided an intuitive assessment of the reliability of the results, while validating the MR assumptions. All analyses were conducted in R v4.2.0 (https://www.R-project.org/), using the TwoSampleMR package, ensuring the standardized implementation of MR methodologies and facilitating reproducibility.

### 2.6. Ethics statement

This study exclusively utilized summary-level data from previously published GWAS, all of which had obtained appropriate ethical approval and participant consent from their original studies. As our analysis involved only de-identified, aggregate genetic data from publicly available sources, additional institutional review board approval was not required per our institutional guidelines and applicable regulations governing the secondary analysis of existing genomic data.

## 3. Results

Our rigorous selection process yielded 64, 40, 65, and 44 independent SNPs that were valid genetic instruments for alcohol intake frequency, coffee consumption, cheese intake, and anxiety, respectively (Table 1). The exclusion of additional SNPs beyond the standard quality thresholds was guided by a comprehensive evaluation of linkage disequilibrium patterns, population stratification effects, and pleiotropic potential, ensuring instrument validity for causal inference.

**Table 1:** Data sources for instrumental variable.

Traits	No. of participants	Ancestry	Number of SNPs available	Number of SNPs used	GWAS ID
Alcohol intake frequency (times/wk)	462,346	European	9,851,867	64	ukb-b-5779
Coffee intake (cups/day)	428,860	European	9,851,867	40	ukb-b-5237
Cheese intake (servings/wk)	451,486	European	9,851,867	65	ukb-b-1489
Seen doctor (GP) for nerves, anxiety, tension, or depression (yes/no)	459,560	European	9,851,867	44	ukb-b-6991

GP = general practitioner, GWAS = genome-wide association studies, SNPs = single nucleotide polymorphisms.

Initial IVW analysis revealed distinct exposure–outcome relationships: while coffee consumption showed no significant association with GERD risk (odds ratio [OR] = 1.21, 95% confidence interval [CI] = 0.98–1.60, *P* > .05), we observed strong causal effects for alcohol intake frequency (OR = 1.52, 95% CI = 1.25–1.84, *P* < .001), cheese consumption (OR = 0.36, 95% CI = 0.26–0.50, *P* < .001), and anxiety-related healthcare utilization (OR = 22.60, 95% CI = 12.12–42.15, *P* < .001).

**Figure 3.**
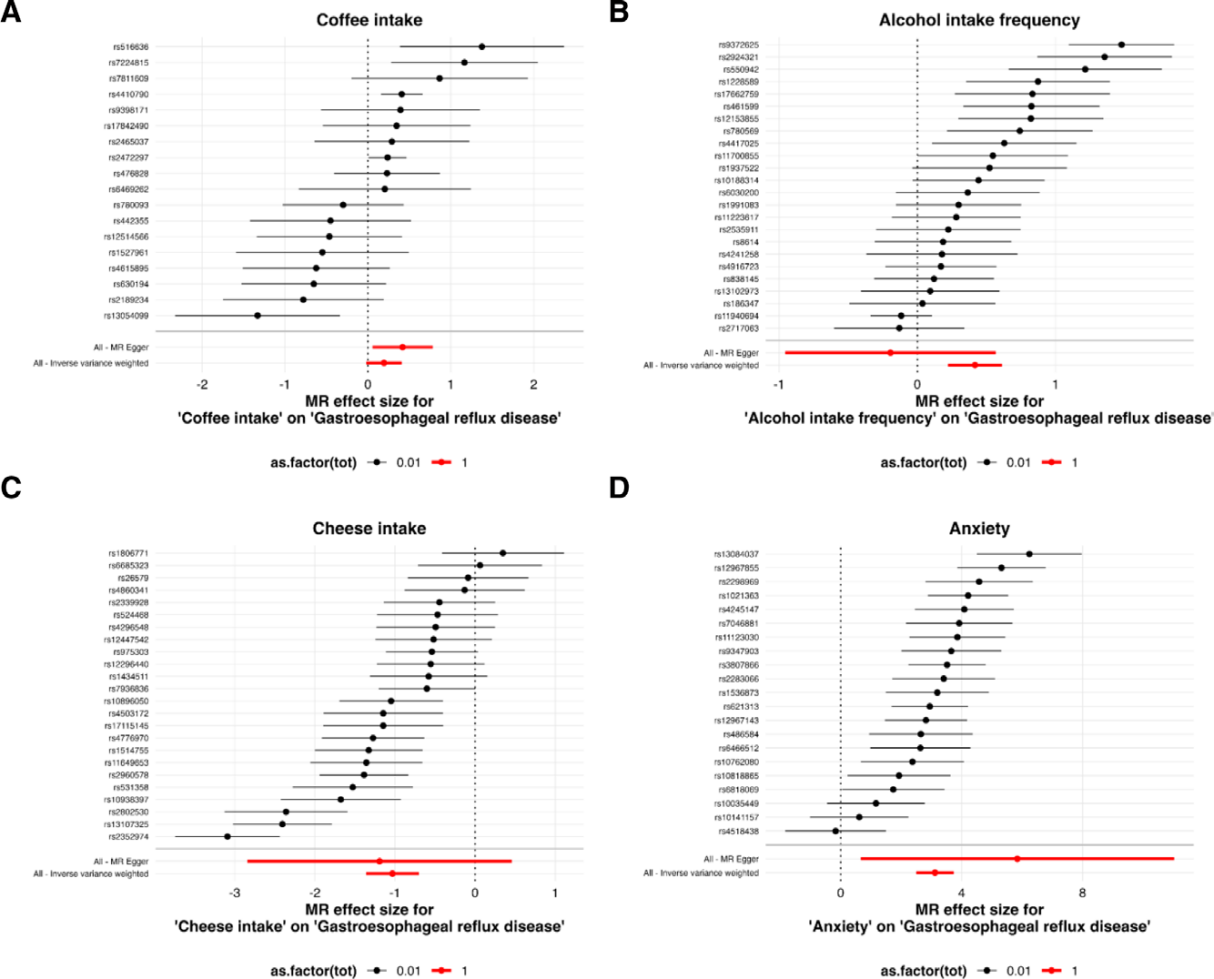
Forest plots of Mendelian randomization analyses for the associations between 4 exposures and gastroesophageal reflux disease. (A) Coffee intake; (B) alcohol intake frequency; (C) cheese intake; (D) anxiety. Forest plots display the causal effects of individual genetic variants and combined estimates for each exposure on GERD risk. Each row represents a SNP used as an instrumental variable. The point estimates (squares) and 95% confidence intervals (horizontal lines) show the association between each SNP and GERD risk. The diamond at the bottom represents the combined IVW estimate with its 95% confidence interval. The size of each square corresponds to the weight of that SNP in the meta-analysis. SNPs are labeled with their respective rs numbers. GERD = gastroesophageal reflux disease, IVW = inverse-variance weighted, SNPs = single nucleotide polymorphisms.

Notably, the association patterns varied across analytical methods. For coffee intake, while the primary IVW analysis was non-significant, the weighted mode method indicated a potential association (OR = 1.35, *P* < .05). Similarly, for alcohol intake frequency, the weighted median approach supported the positive association (OR = 1.24, *P* < .05), though MR-Egger and mode-based methods showed non-significant results. Cheese intake consistently demonstrated a protective association across most methods, in contrast to the positive relationship observed with anxiety. The complete results across all 5 MR methods are presented in Table 2 and illustrated in Figure 3, providing a comprehensive view of the causal relationships through complementary analytical frameworks.

**Table 2:** The results of the MR analysis were conducted to evaluate the potential association between alcohol intake frequency, coffee intake, cheese intake, and the seen doctor (GP) for nerves, anxiety, tension, or depression with the risk of GERD.

Exposure	Methods	OR	95% CI	*P*
Alcohol intake frequency	MR-Egger	0.82	(0.38–1.76)	.621
	Weighted median	1.24	(1.04–1.46)	.013
	Inverse variance weighted	1.52	(1.25–1.84)	<.001
	Simple mode	1.27	(0.92–1.76)	.165
	Weighted mode	1.13	(0.92–1.39)	.243
Coffee intake	MR-Egger	1.52	(1.06–1.50)	.038
	Weighted median	1.30	(1.07–1.57)	.008
	Inverse variance weighted	1.21	(0.98–1.60)	.074
	Simple mode	1.27	(0.77–2.09)	.365
	Weighted mode	1.35	(1.15–2.18)	.002
Cheese intake	MR-Egger	0.30	(0.06–1.58)	.171
	Weighted median	0.56	(0.43–0.72)	<.001
	Inverse variance weighted	0.36	(0.26–0.50)	<.001
	Simple mode	0.59	(0.36–0.96)	.043
	Weighted mode	0.59	(0.38–0.90)	.022
Seen doctor (GP) for nerves, anxiety, tension, or depression	MR-Egger	343.90	(1.93–61157.01)	.040
	Weighted median	18.16	(9.74–33.86)	<.001
	Inverse variance weighted	22.60	(12.12–42.15)	<.001
	Simple mode	29.88	(10.14–88.08)	<.001
	Weighted mode	23.64	(8.20–68.16)	<.001

CI = confidence interval, GERD = gastroesophageal reflux disease, GP = general practitioner, MR = Mendelian randomization, OR = odds ratio, SNPs = single nucleotide polymorphisms.

Our comprehensive sensitivity analysis confirms the robustness of the primary findings. MR-Egger regression detected no significant directional pleiotropy for any exposure–outcome association (all intercepts *P* > .05, Table 3), supporting the validity of the instrumental variables. However, Cochran *Q* test revealed substantial heterogeneity among the genetic instruments for all 4 risk factors (with all *P* < .01, Table 4), which was consistent with the expected biological complexity of these traits. We addressed this heterogeneity using random-effects IVW models,^[24]^ which appropriately weighted individual SNP estimates while accounting for between-variant differences, thereby producing more conservative and reliable effect estimates. The convergence of results across multiple analytical approaches, coupled with a rigorous assessment of assumptions, strengthens the confidence in the causal inferences drawn from this study.

**Table 3:** Results of the multi-effectiveness evaluation of the 4 exposure factors by applying MR-Egger regression.

Exposure	Egger intercept	SE	*P*
Alcohol intake frequency	0.015	0.009	.118
Coffee intake	-0.005	0.003	.161
Cheese intake	0.003	0.013	.848
Seen doctor (GP) for nerves, anxiety, tension, or depression	-0.017	0.017	.313

df = degrees of freedom, GP = general practitioner.

**Table 4:** Results of heterogeneity evaluation of 4 risk factors.

Exposure	Method	*Q*	*Q* df	*Q*_pval
Alcohol intake frequency	MR-Egger	90.67	22	<.001
	Inverse variance weighted	101.56	23	<.001
Coffee intake	MR-Egger	37.22	16	.002
	Inverse variance weighted	42.25	17	<.001
Cheese intake	MR-Egger	125.79	22	<.001
	Inverse variance weighted	126.01	23	<.001
Seen doctor (GP) for nerves, anxiety, tension or depression	MR-Egger	62.37	19	<.001
	Inverse variance weighted	65.90	20	<.001

df = degrees of freedom, GP = general practitioner.

The Figure S1, Supplemental Digital Content, https://links.lww.com/MD/R367 presents scatter plots demonstrating consistent directional associations between all 4 exposure factors (frequency of alcohol consumption, coffee intake, cheese intake, and anxiety) and GERD risk across multiple analytical methods. The robustness of these findings was further confirmed through leave-one-out sensitivity analysis, which revealed that no individual SNP exerted a disproportionate influence on the overall effect estimates (Figure S2, Supplemental Digital Content, https://links.lww.com/MD/R367). Additionally, the funnel plot analysis (Figure S3, Supplemental Digital Content, https://links.lww.com/MD/R367) showed a symmetrical distribution of variant-specific estimates, providing visual confirmation of the absence of significant horizontal pleiotropy.

## 4. Discussion

Our study provides compelling genetic evidence supporting a well-established relationship between psychological factors and GERD development. The robust association observed between anxiety and GERD risk (OR = 22.60, 95% CI: 12.12–42.15, *P* < .001) aligns with and extends previous epidemiological findings, including a large cross-sectional study demonstrating a 2.8-fold increased GERD risk among individuals with anxiety and depression (95% CI: 2.4–3.2).^[32]^ The biological plausibility of this strong association is supported by multiple mechanistic pathways, such as anxiety-induced alterations in esophageal physiology through lower esophageal sphincter relaxation and enhanced visceral sensitivity,^[33]^ neurochemical mediation via cholecystokinin signaling,^[34,35]^ and stress-related changes in gastric acid secretion and digestive motility. Collectively, this mechanistic evidence, together with our genetic findings, underscore the importance of integrating psychological assessments and interventions into GERD management protocols, particularly given the bidirectional relationship between psychological distress and chronic gastrointestinal symptoms.

The considerable magnitude of this genetic odds ratio for anxiety on GERD risk warrants careful interpretation. We posit that this large effect size is directly attributable to our operational definition of the exposure. The genetic instruments were derived from a GWAS of individuals who had “consulted a general practitioner for nerves, anxiety, tension, or depression.” We acknowledge that this constitutes a broad definition, encompassing a spectrum of common, co-occurring psychological complaints in primary care. Paradoxically, however, this very breadth is key to understanding the large effect size. This definition specifically captures a phenotype of significant clinical severity—individuals whose psychological distress (whether categorized as anxiety, depressive symptoms, or related somatic complaints like “nerves” and “tension”) was substantial enough to prompt healthcare-seeking behavior. Consequently, the observed OR likely reflects a particularly strong causal effect within this defined high-risk, clinically relevant subgroup, rather than the effect of milder, subclinical psychological symptoms in the general population. This interpretation is consistent with the principles of MR, wherein analyses using instrumental variables for severe, clinically ascertained exposures can yield larger effect estimates on binary outcomes compared to studies of population-based continuous traits.^[36]^ It is also aligned with other MR studies that have reported large genetic effect sizes (ORs > 10) for severe psychiatric and behavioral phenotypes on various health outcomes.^[37,38]^

The novel inverse association between cheese intake and GERD risk (OR = 0.36, 95% CI: 0.26–0.50) challenges conventional dietary recommendations that often limit high-fat dairy products. While previous observational studies have yielded conflicting results due to methodological limitations,^[39,40]^ our MR analysis provides more reliable evidence by overcoming confounding and reverse causality. The protective effect may be mediated through multiple nutritional components in cheese, including the ability of magnesium to reduce oxidative stress and systemic inflammation,^[41,42]^ the role of calcium in maintaining mucosal integrity, and probiotic modulation of gut immunity.^[43,44]^ These findings suggest that blanket dietary restrictions on cheese may be unwarranted, and highlight the need for more nuanced, evidence-based nutritional guidance for patients with GERD.

For coffee consumption, the primary IVW analysis indicated no significant causal association with GERD risk (OR = 1.21, 95% CI: 0.98–1.60, *P* > .05). Although the weighted mode method suggested a potential positive association (OR = 1.35, *P* < .05), we prioritized the IVW result given its higher statistical power and the absence of directional pleiotropy. Therefore, our MR study does not provide strong genetic evidence to support a causal link. This overall null finding contradicts some observational studies suggesting an increased risk,^[12]^ but aligns with more recent evidence indicating no substantial association.^[45]^ However, these results should be interpreted within the context of the MR design, which investigates lifelong genetic predisposition and may not capture short-term effects of acute coffee intake. It remains possible that other factors, such as specific brewing methods, dosage, or the addition of sugar and milk, could influence reflux symptoms in ways not assessed by our analysis. Nevertheless, the biological plausibility of a null association at the genetic level is supported by the complex composition of coffee, where caffeine may theoretically promote reflux through LES relaxation, while other components, such as chlorogenic acids, may exert protective effects on the esophageal mucosa.

Regarding alcohol consumption frequency, in contrast to the null findings from MR-Egger and mode-based methods, our primary analysis using the IVW method revealed a significant positive causal effect on GERD risk (OR = 1.52, 95% CI: 1.25–1.84, *P* < .001). We prioritized the IVW estimate due to its greater statistical power under balanced pleiotropy, an assumption supported by the non-significant MR-Egger intercept (*P* > .05, Table 3). The discrepancy with some clinical observations^[10]^ might be attributed to residual confounding in those studies, while our genetic approach minimizes such issues. The substantial heterogeneity (Table 4) and the consistency of the positive direction across most methods (Fig. 3, Figure S1, Supplemental Digital Content, https://links.lww.com/MD/R367) strengthen the evidence for a genuine causal effect. The biological plausibility of this finding is well-supported by multiple specific pathways through which alcohol is known to promote gastroesophageal reflux. Firstly, alcohol can provoke transient lower esophageal sphincter relaxations, which are the primary events allowing reflux to occur.^[46]^ Secondly, it directly reduces the resting tone of the lower esophageal sphincter, compromising the primary barrier against gastric content backflow.^[47]^ Beyond its effects on the anti-reflux barrier, alcohol impairs esophageal clearance by diminishing the amplitude of peristaltic waves, leading to prolonged contact between acidic refluxate and the esophageal mucosa.^[48]^ Furthermore, alcohol stimulates gastric acid secretion, thereby increasing the volume and aggressiveness of the refluxate.^[49]^ The convergence of these mechanisms (impairing barrier function, delaying clearance, and enhancing acidity) provides a coherent pathophysiological explanation for the observed genetic causal link between alcohol consumption and GERD.

Collectively, our findings on alcohol, cheese, and anxiety demonstrate significant heterogeneity, as indicated by Cochran *Q* test (Table 4). It is important to contextualize the implication of this heterogeneity for causal inference. In MR, heterogeneity among genetic instrument estimates can arise for several reasons, including the presence of horizontal pleiotropy.^[26]^ However, the non-significant MR-Egger intercept tests (Table 3) make systematic directional pleiotropy an unlikely sole explanation.^[46]^ Instead, the observed heterogeneity most plausibly reflects the inherent biological complexity of the exposures themselves.^[50]^ For instance, genetic variants associated with alcohol consumption frequency may influence GERD risk through a variety of distinct mechanisms beyond a single pathway, such as differential effects on lower esophageal sphincter tone, gastric acid secretion, and mucosal integrity. Similarly, the genetic influences on anxiety and cheese consumption are multifaceted. Therefore, in this study, heterogeneity does not undermine the validity of the primary causal conclusions but rather underscores the multifactorial nature of the relationships we investigated. Our use of random-effects IVW models, which account for heterogeneity by allowing for variation in the variant-specific causal estimates, provides robust evidence for the existence of a net causal effect, even in the presence of such complex underlying biology.^[51]^

Methodologically, this study advances the field through several key strengths. First, our MR design circumvents confounding and reverse causality limitations, plaguing prior observational studies. Second, the application of multiple complementary MR estimators (IVW, MR-Egger, weighted median) with comprehensive sensitivity analyses ensures robust inferences. Third, the focus on European ancestry minimizes population stratification bias while providing substantial statistical power. Most importantly, this represents the first genetic investigation to establish causal cheese–GERD relationships while definitively addressing the coffee–GERD controversy.

Several limitations of this study warrant consideration when interpreting these results. The lifelong exposure perspective inherent in MR analysis may not reflect short-term dietary or psychological interventions. The exclusive focus on European populations limits generalizability to other ethnic groups, particularly given the known variations in lactose tolerance and dietary patterns. Furthermore, our analysis could not differentiate between the types of alcohol and coffee, which may have distinct physiological effects. Future studies should explore these nuances while investigating the molecular mechanisms underlying our observed associations, particularly the potential protective pathways of dairy components in GERD pathogenesis. These findings emphasize the importance of integrating genetic epidemiology with clinical and mechanistic studies to refine GERD prevention and management strategies.

## 5. Conclusion

This MR study strengthened the causal role of anxiety and alcohol intake in GERD etiology and revealed a novel protective association with cheese consumption, while finding no genetic evidence to support a causal role for coffee. These findings advocate for a refined GERD risk model that integrates psychological factors and evidence-based dietary guidance.

## Acknowledgments

We gratefully acknowledge the UK Biobank and QSkin Health Study for providing access to the genome-wide association meta-analysis data used in this study. We extend our sincere appreciation to all participating researchers, study staff, and most importantly, the participants of these cohort studies, whose contributions made this research possible.

## Author contributions

**Conceptualization:** Di Wu.

**Data curation:** Fengyun Guo.

**Formal analysis:** Shengnan Yang.

**Software:** Lijing Bao.

**Visualization:** Ruiying Zhang.

**Writing – original draft:** Qingqing Zhang.

**Writing – review & editing:** Ping Wang.

## Supplementary Material
