# Supplementary material for: Genetic evidence links alcohol, coffee, cheese, and anxiety to GERD risk: A Mendelian randomization study
Source: Medicine (Baltimore). 2026 Feb 20;105(8):e47701. doi: 10.1097/MD.0000000000047701 (PMC12928923; doi:10.1097/MD.0000000000047701)

**Supplementary Figure 1.** Scatter plots of genetic associations with exposures against genetic associations with gastroesophageal reflux disease. (A) Coffee intake, (B) Alcohol intake frequency, (C) Cheese intake, (D) Anxiety. Scatter plots illustrate the relationship between SNP-exposure associations (x-axis) and SNP-outcome associations (y-axis) for each exposure-GERD pair. Each point represents an individual SNP, with error bars indicating standard errors. The slopes of the fitted lines represent the causal estimates from different Mendelian randomization methods: inverse variance weighted (blue line), MR-Egger (red line), weighted median (green line), weighted mode (orange line), and simple mode (light green line).


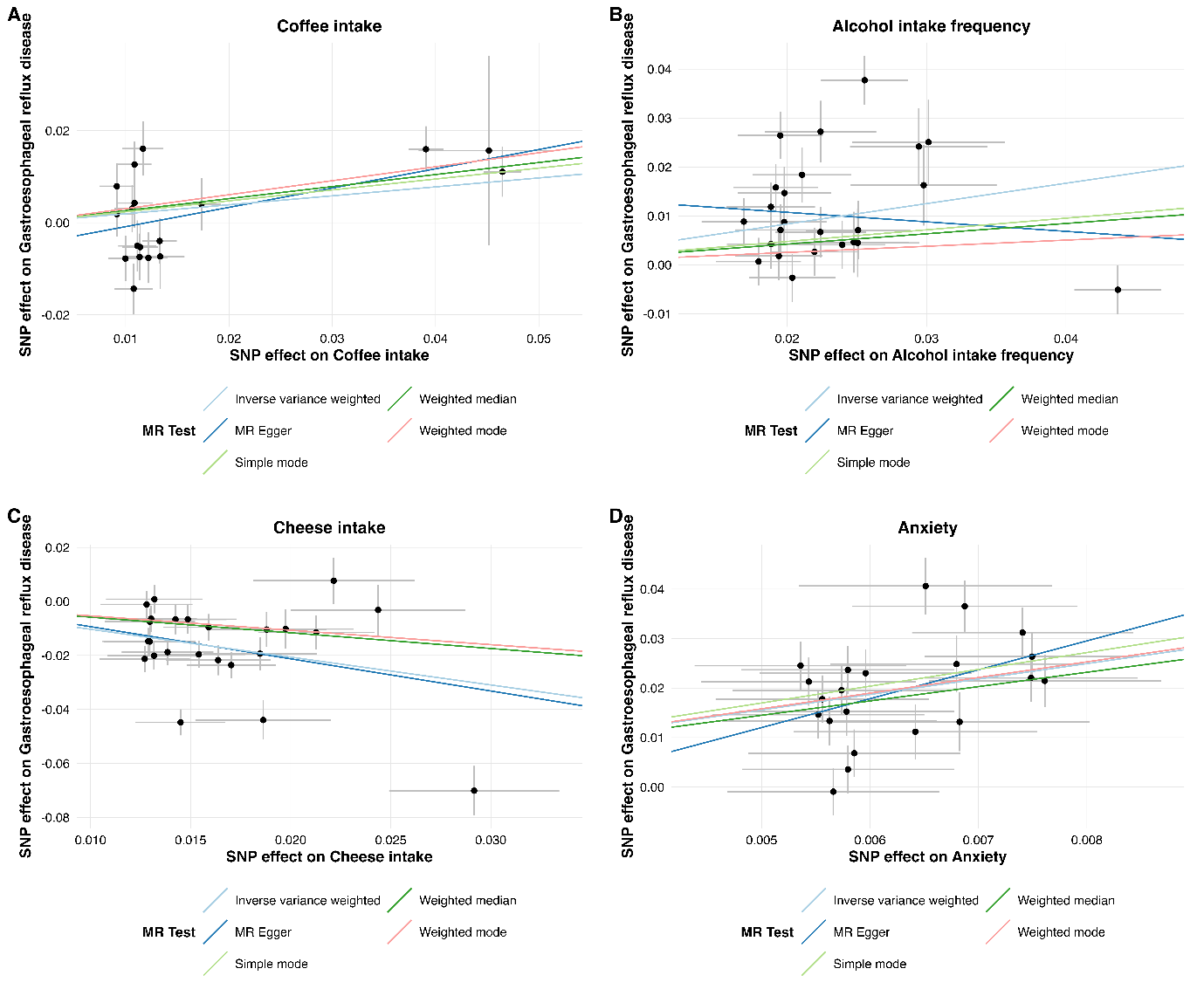


**Supplementary Figure 2.** Leave-one-out sensitivity analysis for Mendelian randomization estimates. (A) Coffee intake, (B) Alcohol intake frequency, (C) Cheese intake, (D) Anxiety. Leave-one-out analysis demonstrates the influence of individual SNPs on the overall causal estimate. Each row shows the IVW estimate when that particular SNP is excluded from the analysis. The red vertical line indicates the combined IVW estimate using all SNPs. The stability of causal estimates across different leave-one-out iterations suggests that no single SNP is driving the observed associations.


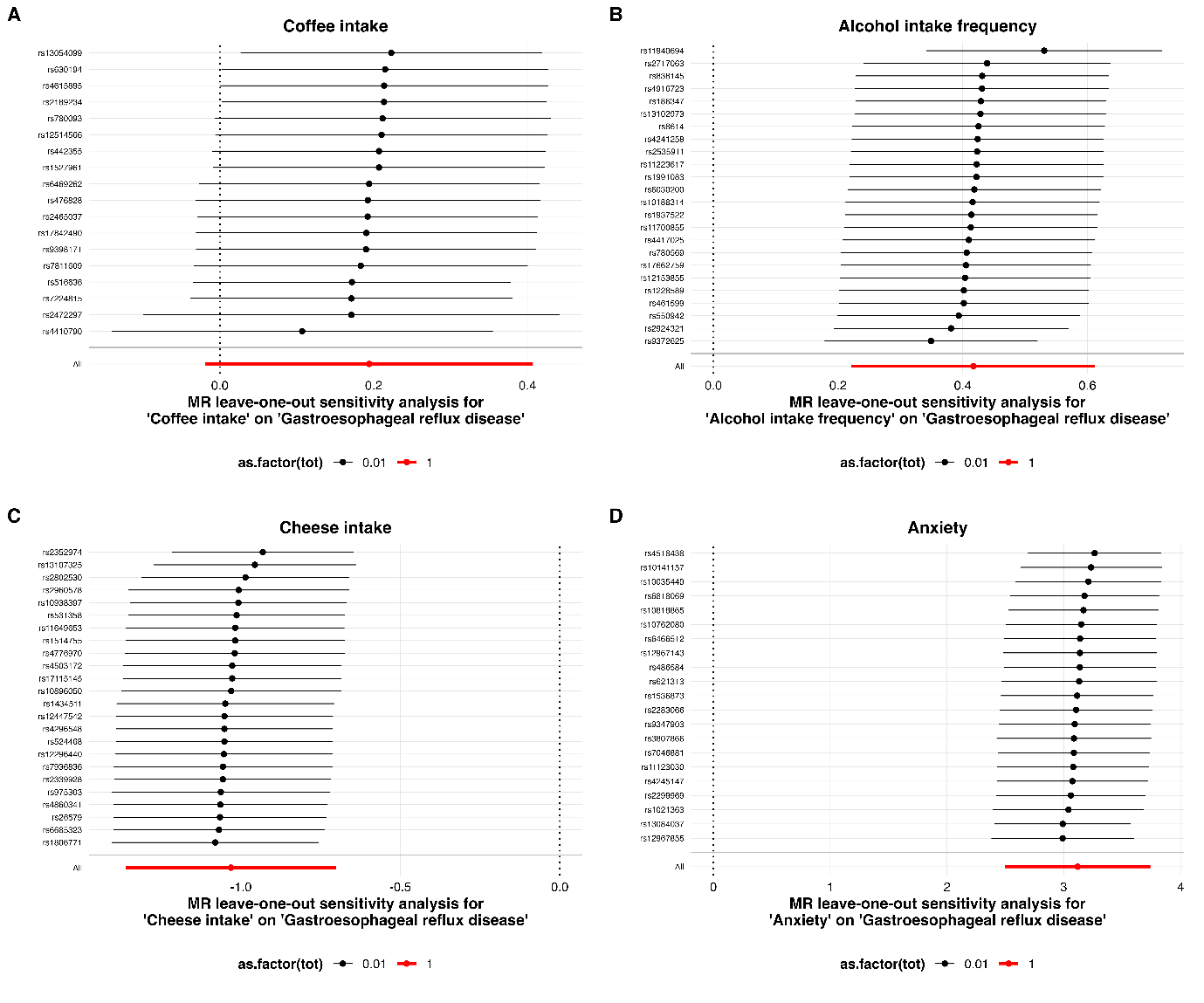


**Supplementary Figure 3.** Funnel plots assessing potential directional pleiotropy in Mendelian randomization analyses. (A) Coffee intake, (B) Alcohol intake frequency, (C) Cheese intake, (D) Anxiety. Funnel plots evaluate the symmetry of SNP-specific estimates around the overall causal estimate to detect potential directional pleiotropy. Each point represents an individual SNP, plotted by its precision (1/standard error, y-axis) against its causal estimate (x-axis). In the absence of significant pleiotropy, the points should be symmetrically distributed around the combined IVW estimate. Asymmetry in the funnel plot may indicate the presence of directional pleiotropy, which could bias the causal estimates. The dashed lines represent the 95% confidence intervals. Light and dark green indicate reliable estimates from the IVW and MR-Egger methods, respectively.


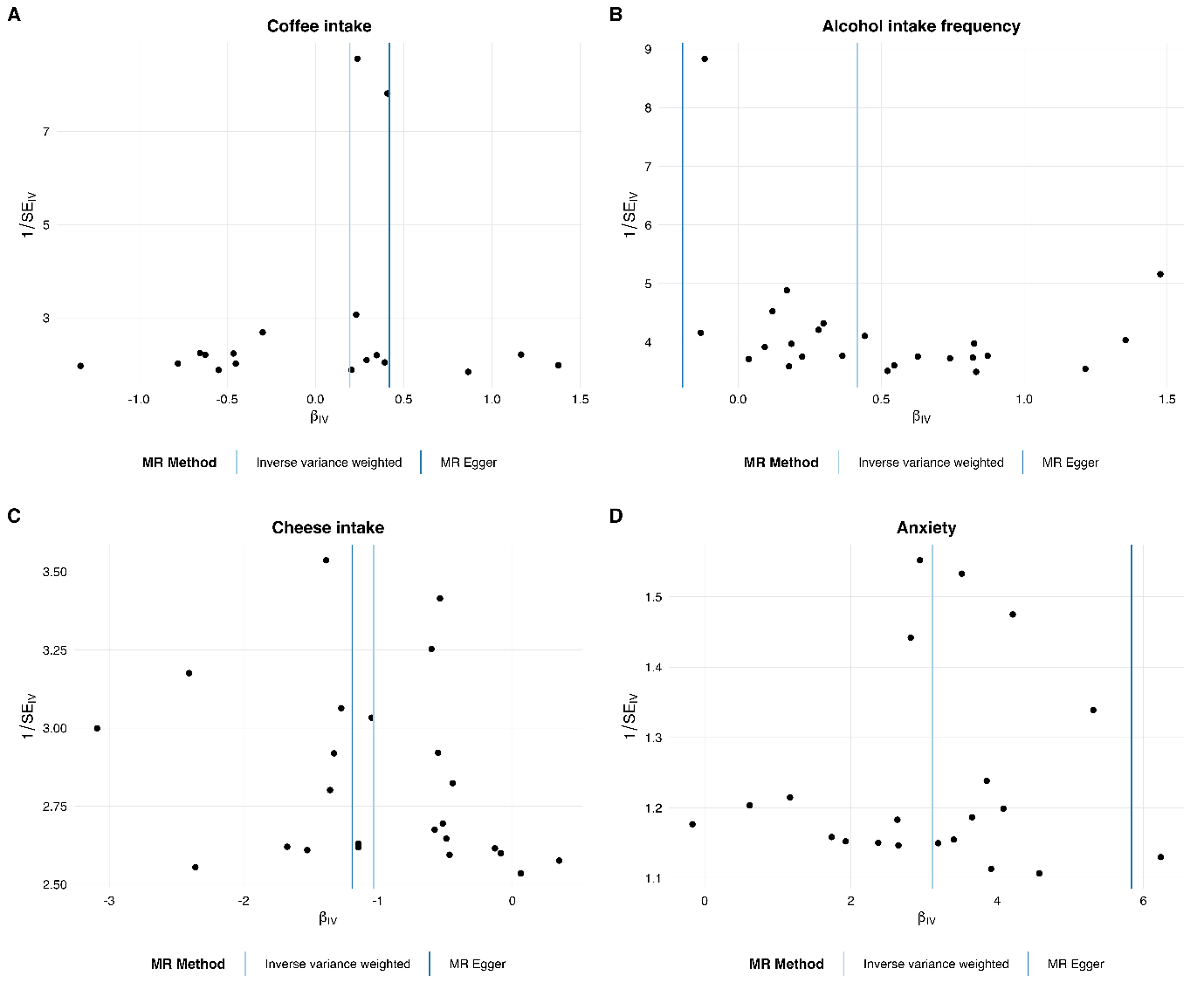

Supplement: Supplementary file 1 [file medi-105-e47701-s001.docx]
